# Kinase-Targeted Therapies for Glioblastoma

**DOI:** 10.3390/ijms26083737

**Published:** 2025-04-15

**Authors:** Maria Salbini, Alessia Formato, Maria Patrizia Mongiardi, Andrea Levi, Maria Laura Falchetti

**Affiliations:** Institute of Biochemistry and Cell Biology, National Research Council, Via Ercole Ramarini 32, Monterotondo, 00015 Rome, Italy; mariasalbini@cnr.it (M.S.); alessiaformato@cnr.it (A.F.); mariapatrizia.mongiardi@cnr.it (M.P.M.); andrea.levi@cnr.it (A.L.)

**Keywords:** glioblastoma, therapy, kinase inhibitor

## Abstract

Protein phosphorylation and dephosphorylation are key mechanisms that regulate cellular activities. The addition or removal of phosphate groups by specific enzymes, known as kinases and phosphatases, activates or inhibits many enzymes and receptors involved in various cell signaling pathways. Dysregulated activity of these enzymes is associated with various diseases, predominantly cancers. Synthetic and natural single- and multiple-kinase inhibitors are currently being used as targeted therapies for different tumors, including glioblastoma. Glioblastoma IDH-wild-type is the most aggressive brain tumor in adults, with a median overall survival of 15 months. The great majority of glioblastoma patients present mutations in receptor tyrosine kinase (RTK) signaling pathways responsible for tumor initiation and/or progression. Despite this, the multi-kinase inhibitor regorafenib has only recently been approved for glioblastoma patients in some countries. In this review, we analyze the history of kinase inhibitor drugs in glioblastoma therapy.

## 1. Introduction

Protein phosphorylation/dephosphorylation processes, performed by a specific class of proteins named kinases and phosphatases, respectively, regulate a wide range of biological processes, often as a response to external stimuli [1,2,3]. Protein phosphorylation is the most common post-translational modification and represents an extremely potent tool to enhance protein function. More than 500 protein kinases have been identified so far, which add a phosphate group from ATP to target proteins, eventually turning them on [1,4]. Concerning the targeted protein residue, kinases might be categorized as serine, threonine, or tyrosine kinases. Most kinases target both serine and threonine, while some specifically target tyrosine. Some kinases can act on all three [5,6]. A significant percentage of human protein is a kinase target and the majority of cellular pathways, including those related to signal transduction, are regulated by protein kinases [7]. Fundamental cellular processes, like protein synthesis, cell division, signal transduction, cell growth, development, and aging, are finely regulated by protein phosphorylation. Failure of kinase activity might result in dramatic changes in these biological processes. Protein kinase misregulation might be caused by genetic mutations and can be responsible for the pathogenesis of different diseases, ranging from inflammatory and cardiovascular diseases to cancer. Key events of cancer, like tumorigenesis, progression, invasion, and metastasis, are driven by deregulated protein kinases. The role of altered kinase activity in different neoplasms has been widely addressed [8], and the study of anti-kinase molecules has become central in the anti-cancer research of the 21st century (for recent reviews see [9,10]), including those on glioblastoma [11,12,13]. Kinase inhibitors (KIs) come in different flavors and are generally classified into five different classes or types (although other authors distinguish up to seven different types) [14].

**Figure 1:**
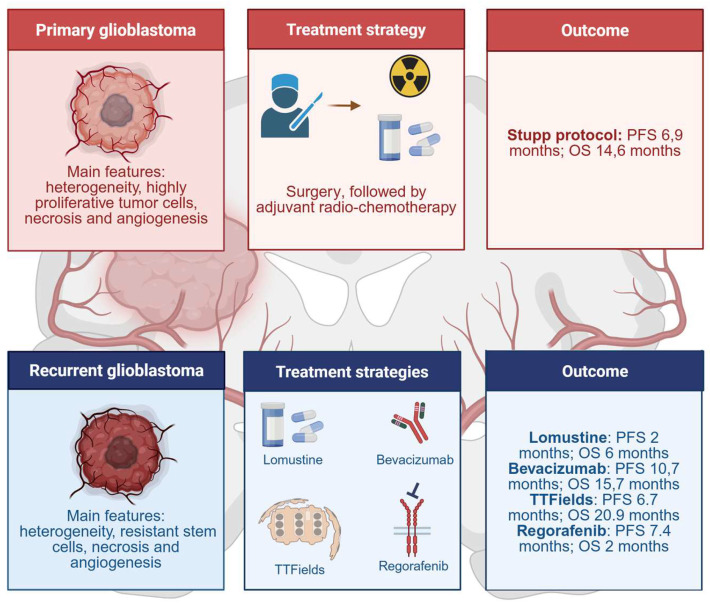
Histopathological features of glioblastoma and current therapies. Primary glioblastoma is characterized by large heterogeneity, high cellular proliferative index, wide necrotic areas, and massive angiogenesis. Standard therapy is based on massive surgery followed by adjuvant radiation and chemotherapy with the alkylating agent temozolomide. The presence of therapy-resistant glioma stem cells (GSCs) characterizes recurrent glioblastoma. Therapeutic options for relapsed tumors are poor.

Type 1 inhibitors are small molecules that bind to kinases in their active conformation and compete for the binding of ATP, thus preventing kinase-mediated phosphorylation of targets. Many FDA-approved kinase inhibitors belong to this class; however, since the ATP binding site is conserved among different kinases, type 1 inhibitors often have low selectivity and may cause off-target complications (see [15]). Class 2 inhibitors bind to kinases in their inactive conformation in a hydrophobic region near the ATP binding site and keep the kinases in their inactive conformation. They are generally more selective than class 1 inhibitors [16]. Type 3 inhibitors regulate the activity of their target kinase via an allosteric mechanism and bind far from the catalytic domain/ATP binding site (sometimes type 3 inhibitors are divided into two subtypes, one binding near and the other far from the ATP binding region). They are potentially quite selective since their binding sites may be specific for each kinase. Furthermore, they may selectively target mutant forms of kinases, sparing normal ones [17]. Type 4 kinase inhibitors, also known as substrate-directed inhibitors, prevent the interaction between kinases and their targets [18]. Type 5 inhibitors, also known as covalent kinase inhibitors, form a stable complex with a cysteine present in the ATP binding site. Type 5 inhibitors tend to have high potency and extended pharmacodynamics [19,20]. Today, there are 80 FDA-approved drugs targeting more than 20 different protein kinases. The great majority of these 80 molecules are approved for cancer treatment (69/80) [21] (Figure 1).

The https://www.icoa.fr/pkidb/ (accessed on 10 April 2025) website provides an updated list of protein kinase inhibitors in clinical trials. There are currently 434 molecules under evaluation. The first kinase inhibitor approved for clinical use by the FDA was imatinib (Gleevec) in 2001 [22]. This drug transformed an almost incurable cancer, chronic myeloid leukemia, into a manageable condition, paving the way to multi-kinase inhibitor development for cancer therapy.

When considering kinase inhibitors as a possible therapeutic strategy for cancer, a basic dilemma is as follows: should we focus on a single-kinase inhibitor or alternatively on multi-kinase inhibitors (MKIs)? In other words, as Broekman and colleagues stated in their 2011 review [23], which strategy might be more effective, the use of inhibitors with multiple targets or, alternatively, the use of several single inhibitors? What’s better, single–multi or multi–single? Almost fourteen years later, the question is still open. Several issues should be considered when choosing the strategy between a drug targeting a single kinase, to be used in combination with other inhibitors, and a multi-target kinase inhibitor. Both multi-kinase inhibitors and single-kinase inhibitors come with their own set of benefits and drawbacks, which are linked to possible resistance mechanisms, pharmacokinetics, selectivity, and tumor microenvironment. Various tyrosine kinases are either mutated or overexpressed in different cancers, leading to several resistance pathways. Pharmacokinetics can vary significantly among individuals, and differences are observed between patients treated with the same tyrosine kinase inhibitor or with two different targeted inhibitors. Each tyrosine kinase inhibitor employs distinct mechanisms to achieve selectivity, and there are differences in gene expression between tumor cells and stromal cells. Given these factors, the choice between multi-kinase and single-kinase inhibitors cannot be universally preferred but must be tailored to the patient’s unique genetic profile and the specific characteristics of their tumor, enabling personalized therapy. As a further consideration, the efficiency of kinase inhibition of each multi-kinase inhibitor may produce quite a different result according to the target [24].

**Figure 2:**
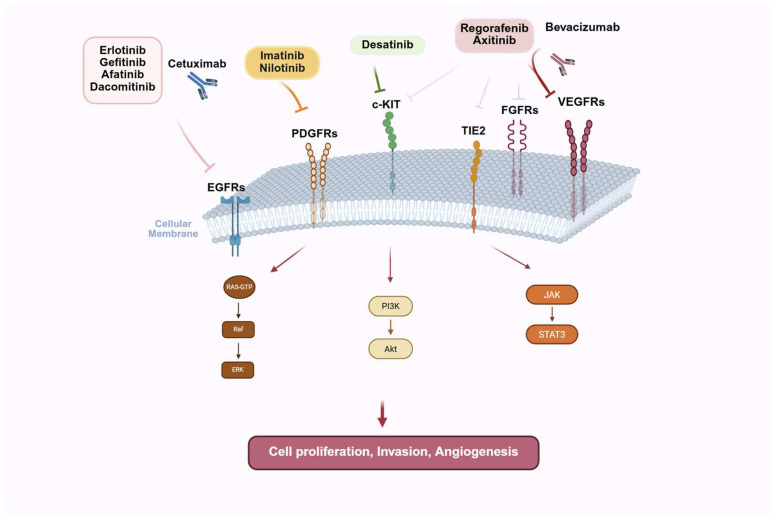
Kinase receptors and their inhibitors. Kinase inhibitors target different receptors affecting multiple cellular pathways involved in the regulation of key biological processes.

Glioblastoma IDH-wild-type, previously referred to as glioblastoma multiforme in accordance with its peculiar intra- and inter-tumoral heterogeneity, is the most aggressive form of brain tumor in adults [25]. It is characterized by highly proliferative tumor cells, extended necrotic areas, and prominent angiogenesis. The clinical management of the disease is substantially based on surgery, followed by adjuvant radio-chemotherapy based on the alkylating agent temozolomide. Due to its highly invasive way of growth, surgery does not allow for complete tumor removal, and early relapses are the standard *iter* of the disease. Clinical options for recurrent patients are scarce (Figure 2). Lomustine treatment is the standard approach, but the prognosis is unfortunately dismal [26]. The median survival at diagnosis is only 15 months, and the 5-year relative survival rate is only 6.8% [27,28].

Novel approaches for glioblastoma therapy have been proposed in the last few years. Some of these are focused on the wide tumor-induced angiogenesis, which characterizes glioblastoma. Bevacizumab, a humanized monoclonal antibody targeting vascular endothelial growth factor (VEGF), was approved by FDA for relapsed glioblastoma in 2007 [29]. Unfortunately, bevacizumab has been shown to be less effective in the clinical practice than expected, mainly because of the induction of an enhanced infiltrating phenotype [30,31,32]. In 2011 and 2015, the FDA approved the use of tumor treatment fields (TTFields) for relapsed and primary glioblastoma patients, respectively [33]. This device, applied to the patient’s scalp, delivers low-intensity alternating electric fields which interfere with microtubule polymerization during mitosis and eventually result in tumor cell growth inhibition [34]. Although encouraging results were reported [35], the benefits of TTFields are still debated, and their use was not included in the standard of care. Additionally, the device is hard for patients to bear and its cost is high [36].

In the frame of antiangiogenic approaches, small drugs working as protein kinase inhibitors have been evaluated. To our knowledge, the only multi-kinase inhibitor approved so far for glioblastoma patients is regorafenib (Stivarga), which has been introduced for relapsed patients in Italy following the encouraging results of the randomized, multi-center, open-label phase II trial REGOMA [37]. This review focuses on kinase inhibitors in glioblastoma therapy.

## 2. Kinase Inhibitors: Mechanisms and Therapeutic Approaches

### 2.1. Mechanisms of Action of Kinases

The protein kinase family, also referred to as the kinome, is one of the biggest protein families encoded by the human genome. More than 500 protein kinases (538) belong to this family and about 2% of the human genome encodes protein kinases [1]. The activity of protein kinases is tightly modulated, allowing them to act as molecular switches that are regulated by interactions with other proteins and by post-translational modifications. Protein kinases work by transferring the gamma-phosphate of ATP to the alcohol groups (on Ser and Thr) or phenolic groups (on Tyr) of proteins, eventually producing phosphate monoesters. The addition of the phosphate group to target proteins results in the change in their stability, intracellular localization, and activity. In 1991, a seminal paper was published that presented the first crystal structure of a protein kinase, cyclic-AMP-dependent protein kinase (PKA). This structure revealed that protein kinases share a conserved structural core, consisting of an N-lobe with a five-stranded β-sheet (β1–β5) and at least one α-helix, and a C-lobe that is primarily α-helical, but includes a small yet significant β-sheet (β6–β7) [38]. Protein kinases are activated by phosphorylation which in turn activates a cascade of events leading to the phosphorylation of different amino acids. Due to their key regulatory role in fundamental cellular processes, the alteration of kinase activity is involved in the genesis of different pathologies, ranging from cancer to metabolic, cardiovascular, and immunological diseases. This is the reason why protein kinase inhibitors are widely studied for their potential in clinical therapy.

### 2.2. Multi-Kinase Inhibitors

Several mutated kinase pathways have been extensively characterized in glioblastoma. Data from whole-exome sequencing revealed that at least one RTK is mutated in 67% of glioblastoma patients [39]. Epidermal growth factor receptor (EGFR) is the most frequently mutated receptor in glioblastoma (57% of cases), followed by platelet-derived growth factor receptor (PDGFRA) (13%), fibroblast growth factor receptor (FGFR) (3.2%), and c-MET (1.6%) [40,41,42]. Several pharmacological inhibition strategies have been developed to target kinase activity as an anti-cancer therapy. The following paragraphs examine the most important ones and their targeting in glioblastoma.

#### 2.2.1. Inhibitors of EGFR Kinase Activity

EGFR, also known as HER1 or ErbB1, is a transmembrane receptor with a MW of 170 kDa [43]. It belongs to a family of receptors together with three other members: ErbB2, mErbB3, and ErbB4 [44]. The EGFR monomer is inactive. When it binds its ligand on the extracellular domain, it undergoes dimerization, intracellular kinase domain activation, and downstream targets phosphorylation. EGFR can either homo- or heterodimerize with other receptors of the same family. Besides epidermal growth factor (EGF), EGFR binds transforming growth factor alpha (TGF-α) and heparin-binding EGF-like growth factor (HB-EGF). The downstream effectors of EGFR include MAPK (mitogen-activated protein kinase), PI3K-AKT, SRC, PLC-γ1-PKC, JNK, and JAK-STAT pathways [45,46]. Heterodimerization with other ErbB family members allows it to interact with a wider number of targets.

As stated before, EGFR is the most frequently mutated protein in human glioblastoma. The most common EGFR mutation in glioblastoma patients is the EGFR gene amplification, which occurs in about 40% of cases [47] and is mainly found in primary glioblastomas. Structural aberrations occur as well; the most frequent is the in-frame deletion of exons 2-7, known as EGFRvIII, which results in the lack of a binding domain and constitutive activation. Kinase activity of EGFRvIII is weaker than that of the wild-type receptor, but it is constitutive, eventually resulting in enhanced tumorigenesis and cancer progression [42,48]. Pro-tumorigenic activity of EGFRvIII is due to an improved ability to promote key processes for tumor development, such as cell survival, invasion ability, stemness maintenance, and angiogenesis. EGFRvIII signaling is both qualitatively and quantitatively different from that of the wild-type receptor. Glioblastoma patients with EGFRvIII are characterized by the worst prognosis [49].

Given the frequency of EGFR mutations in human tumors, EGFRs have been proposed as a possible target for anti-cancer therapy. EGFR tyrosine kinase inhibitors (TKIs) have proven to be effective in non-small-cell lung cancer (NSCLC), where they are the major first-line therapy for patients with activating EGFR mutations. The most common EGFR mutations in NSCLC are alterations in the intracellular tyrosine kinase domain. Therapies aimed at these mutations are among the first successful attempts in targeting EGFRs. Erlotinib and gefitinib, two 4-anilinoquinazolines, belong to this category and were successfully used to inhibit downstream EGFR signaling in NSCLC [50,51]. Both of them target signal transduction by binding to the TK domain and inhibiting its activity. Overall, first-generation drugs are competitive inhibitors of ATP binding (type 1 inhibitors). Unfortunately, clinical experience demonstrated that cancer cells rapidly evolve strategies to bypass drug effectiveness and acquire resistance [52]. Second-, third-, and fourth-generation TKIs were therefore developed for NSCLC, in a constant effort to fight cancer cells’ escape mechanisms. Second-generation TKIs, as afatinib and dacomitinib, also target and irreversibly inhibit ErbB2, ErbB3, and ErbB4, but their clinical effectiveness is strikingly reduced by the induction of heavy adverse effects, mainly due to their concomitant inhibitory function on wild-type EGFRs in normal tissue [53]. Since acquired resistance to therapy is highly frequent, third and fourth-generation TKIs work by targeting mutations responsible for therapy resistance. In particular, third-generation drugs target the T790M mutation, which is the cause of about 50% of cases of acquired resistance [54]. Fourth-generation drugs generally target the T790M/C797S EGFR mutant, the leading cause of resistance to third-generation drugs [55]. Overall, the use of TKIs targeting EGFRs in lung cancer has been characterized by a good response, which, unfortunately, does not hold true for glioblastoma. Experiments in preclinical models of glioblastoma, both in vitro and in xenografted mice, were promising [56,57]. However, trials with erlotinib and gefitinib did not provide significant clinical effectiveness. Erlotinib failed to prolong overall survival (OS), either as a single treatment or as a combination treatment. Erlotinib administered with the tyrosine kinase inhibitor sorafenib resulted in a median overall survival of 5.7 months. Progression-free survival at 6 months was 14%. Similarly, the concomitant use of erlotinib and of the VEGF-targeting monoclonal antibody bevacizumab in a phase II trial on relapsed glioblastoma patients did not improve progression-free survival when compared to patients treated with bevacizumab as a monotherapy [58,59]. Gefitinib, although able to dephosphorylate EGFRs in surgical specimens of glioblastoma patients, slightly improved OS, probably because it did not suffice to halt tumor cells’ signaling [60]. These disappointing results were likely dependent on the mechanism of action of erlotinib and gefitinib, which were designed to target the intracellular tyrosine kinase domain, while the most frequent EGFR mutations in glioblastoma affect the extracellular domain. Lapatinib, which is directed against the extracellular domain, had slightly better clinical results in a phase I/II clinical trial [61]. Probably the main reason for erlotinib, gefitinib, and lapatinib having poorer success in glioblastoma than in NSCLC is that they all target the active conformation of EGFRs, while in glioblastoma, EGFRs have an active state despite their inactive conformation [56].

A possible strategy to overcome glioblastoma resistance to TKIs is the use of combination therapy. A series of evidence addresses the improved efficacy of EGFR inhibitors in limiting glioblastoma tumor growth when used together with radiation [62], or with a histone deacetylase inhibitor [63], or with the mTOR inhibitor rapamycin [64].

Other approaches have been evaluated to target EGFRs, based on the use of monoclonal antibodies. Cetuximab blocks the ligand binding domain of the receptor, preventing ligand-triggered dimerization and activation of the kinase activity, eventually resulting in failure to activate downstream pathways. Although cetuximab disappointed the expectations as a monotherapy, the concomitant use of cetuximab with the humanized monoclonal antibody bevacizumab (which targets VEGF) or with irinotecan, a drug belonging to the camptothecin family of topoisomerase I inhibitors, resulted in improved results when compared to single therapy with cetuximab, but therapy efficacy was not improved when compared to bevacizumab or irinotecan alone [65]. In addition, one main problem of cetuximab therapy is the adverse side effects induced on non-cancer cells. Toxicity is also due to the necessity to use high cetuximab dosage, required for allowing effective concentration in the brain following crossing of the blood–brain barrier (BBB). BBB permeability is indeed a key issue for brain tumor targeting.

Cetuximab was also tested with success as cetuximab-CCR5 fusion protein transduced by an oncolytic virus in preclinical models. The idea was to add to the cetuximab-mediated inhibition of EGFRs the immune cell infiltration promoted by CCR5. Oncolytic viruses can be engineered to replicate in tumor cells, induce their lysis, and stimulate an immune response in the tumor microenvironment [66].

#### 2.2.2. Inhibitors of Platelet-Derived Growth Factor (PDGF) Kinase Activity

The signaling of platelet-derived growth factor (PDGF) plays a crucial role in the formation of gliomas and is the main driver of the proneural subtype of glioblastoma [67]. The effects of PDGF signaling are diverse, including stimulating cancer cell growth through autocrine mechanisms and influencing neighboring stromal and vascular cells through paracrine effects. The two PDGFRs, PDGFRα and PDGFRβ, may either homo- or heterodimerize. Ligand binding, according to a mechanism similar to the one reported above for EGFR, induces receptor dimerization, activation of the kinase domain, and phosphorylation of targets on specific tyrosine residues, eventually resulting in the activation of downstream effectors, such as the RAF/MAK and PI3K/AKT pathways.

PDGFRβ is present in more than 90% of endothelial cells in glioblastoma samples. PDGFRα is mutated in about 13% of glioblastoma tumor cells. Several small molecules have been developed that inhibit the kinase activity of PDGFRs, but none of them are characterized by a high specificity. Despite the frequent alterations in PDGF signaling in glioblastoma, no encouraging results have been achieved so far targeting its kinase activity. Clinical trials on imatinib, dasatinib, and nilotinib all resulted in low effectiveness [68,69] (https://clinicaltrials.gov/study/NCT02626364 (accessed on 10 April 2025)). 

### 2.3. Promising Multi-Kinase Inhibitors

Axitinib is a multi-kinase inhibitor with high specificity for vascular endothelial growth factor receptors 1, 2, and 3 (VEGFR1-3), where it exerts its inhibitory activity at picomolar concentrations. At higher doses, in the nanomolar range, axitinib might inhibit other tyrosine kinases [70]. The EMA and FDA first approved axitinib for recurrent metastatic renal cell carcinoma [71], then for metastatic colorectal cancer [72]. Due to its specificity for VEGFRs, it is considered an antiangiogenic drug and, as such, it was tested as a potential therapy for glioblastoma. When tested in orthotopic glioblastoma xenografts in rodents, axitinib demonstrated good efficacy in inhibiting tumor growth and angiogenesis [73]. Unfortunately, its effectiveness as a monotherapy was not confirmed in clinical trials [74,75]. On the other hand, axitinib was associated with an immunostimulatory function in patients with recurrent glioblastoma [76]. Remarkably, a recent paper supports the data that axitinib might improve the immune system response in in vivo preclinical models. It was indeed demonstrated that axitinib, used in combination therapy with the tricyclic antidepressant imipramine in glioblastoma xenografts, is able to improve the prognosis, mainly potentiating an immune response [77].

Axitinib might be responsible for undesired side effects, which are at least in part due to the induction of cellular senescence [78,79,80]. The issue of whether therapy-induced senescence (TIS) is detrimental for anti-cancer therapy or contributes to therapy effectiveness is still open, but a further key aspect deals with toxicity in normal tissues. We demonstrated that the mechanism of axitinib-triggered senescence in glioblastoma cellular models in vitro (bulk tumor cells and patient-derived glioblastoma stem cells—GSCs) is dependent on the intracellular accumulation of reactive oxygen species (ROS) and the activation of the ATM (ataxia telangectasia mutated) kinase. If we administer axitinib together with the antioxidant molecule N-Acetyl-Cysteine (NAC) to both in vitro and in brain orthotopic tumors established by GSCs, we prevent senescence induction, therefore reducing axitinib adverse effects with no impairment of axitinib anti-tumor activity [81]. Overall, taking into account both the indications about the increased axitinib effectiveness in combinatorial therapies and the possibility of lowering axitinib toxicity by the use of antioxidants, axitinib represents a good candidate for glioblastoma therapy.

Regorafenib (BAY 73-4506) is an oral multi-kinase inhibitor targeting a plethora of receptors involved in different biological processes. Among its main targets are FGFRs, PDGFRs, VEGFRs, Tie-2, and the oncogenic kinases RET and KIT. The molecular structure of regorafenib is similar to that of sorafenib, but its pharmacological activity is improved. Regorafenib was firstly approved for the clinical management of advanced colorectal carcinomas, advanced gastrointestinal stromal tumors (GISTs), and advanced hepatocellular carcinomas [82,83,84]. A few years later, regorafenib was approved as a second line treatment by the Italian medicine agency, AIFA, for relapsed glioblastoma patients. Up to now, regorafenib still has not received FDA approval, although it was introduced as a preferred regimen for recurrent glioblastoma by the National Comprehensive Cancer Network (NCCN) 2020 Guideline. The introduction into clinical use, although limited to Italy, was the consequence of a phase II trial, REGOMA, which demonstrated an overall survival benefit in the regorafenib arm of the trial when compared to the lomustine arm [37]. While we are writing this review, there are four completed, two active which are not recruiting, and three completed clinical trials on regorafenib in glioblastoma (according to clinicaltrials.gov website). Two of these studies included or enrolled selectively newly diagnosed glioblastoma patients (designated as NCT03970447 and NCT06095375, respectively), in an attempt to evaluate multiple therapies in newly diagnosed glioblastoma (NCT03970447) or to obtain insights into regorafenib effectiveness in combination with temozolomide, with or without concomitant radiation, in newly diagnosed MGMT-methylated patients (NCT06095375). The clinical experience accumulated over these few years of regorafenib use in glioblastoma therapy has demonstrated that the clinical response is very heterogenous and that stratification criteria allowing pre-selection of responder patients are completely missing. This is due to the absence of a deep characterization of the molecular mechanisms governing glioblastoma cells’ response to regorafenib. The REGOMA team published a paper identifying a mini signature of sensitivity to regorafenib. The study was conducted on surgical specimens from the first surgery of patients enrolled in the REGOMA trial. The authors identified the upregulation of two genes, HIF-1α (hypoxia inducible factor 1α) and CDKN1A (cyclin dependent kinase inhibitor 1A), and the downregulation of three miRNAs, miR-93-5p, miR-3607-3p, and miR-301a-3p, as positive predictive markers of sensitivity to regorafenib [85]. A second omic study was developed by NGS on FFPE (formalin-fixed paraffin embedded)-tumor specimens. As in the case of the previous paper, tumor specimens were derived from first surgery, before regorafenib administration at tumor relapse. In this cohort of 30 patients, a positive correlation between mitogen-activated protein-kinase pathway mutation and low sensitivity to regorafenib was found [86]. A metabolic clue linking glioblastoma cells susceptibility with an energetic metabolism was provided by Indraccolo and colleagues [87], who demonstrated that the activation of the kinase AMPK correlates with sensitivity to regorafenib. They analyzed immunohistochemistry and digital pathology tumor specimens derived from relapsed glioblastoma, either regorafenib treated or not, and hypothesized that regorafenib treatment induced AMPK activation with metabolic reprogramming, eventually leading to an impairment of cell proliferation.

Regorafenib’s mechanism of action has also been investigated in preclinical in vitro and in vivo models, where the main findings can be summarized as follows: regorafenib induces cell death when administered to either bulk (stable) or cancer stem glioblastoma cells. The mechanisms of cell death induction have not been completely elucidated. Although apoptosis has been detected in regorafenib-treated cells, it seems not to be the preferred way of death induction [88,89]. A prevalent mechanism of regorafenib-triggered cell death is autophagy [90]. Regorafenib works by directly stabilizing PSAT1 (phosphoserine aminotransferase 1), a key enzyme involved in the production of serine. This action triggers autophagy through PRKAA (protein kinase AMP-activated catalytic subunit alpha) activation and impairs autophagosome–lysosome fusion, synergistically causing autophagosome accumulation and growth arrest of glioblastoma cells.

The first paper to systematically address the molecular mechanisms of glioblastoma cells response to regorafenib was published at the beginning of 2025 [91]. Here, authors used a CRISPR/Cas9 functional genomics target discovery with activation and knockout screens to identify targets that alter the therapeutic effectiveness of regorafenib in experimental glioma models. Through this experimental approach, they identified a series of regorafenib response modifiers, including BCL2, BCL2L1, ITGB3, FOXC1, SERAC1, ARAF, and PLCE1. In particular, BCL2 and BCL2L1 were upregulated upon regorafenib treatment. The concomitant use of regorafenib and navitoclax, an inhibitor of Bcl2 family members, showed a synergistic anti-tumor effect both in vitro and in xenograft tumor models established by injecting stable glioma cell lines or glioma stem-like cells. The main limitation of the study was the cellular models in which the CRISPR/Cas9 screens were performed, that is the established (bulk) glioblastoma cell lines. It is well known, indeed, that this cellular model has intrinsic limitations and that tumor stem cells better recapitulate the histopathological characteristics of the parental tumor and represent the tumor cell subpopulation mainly responsible for therapy resistance. Notwithstanding this limitation, the experimental approach adopted in this paper allows for the fine molecular analysis of the molecular mechanisms at the foundation of tumor cell response to the drug and allows for the identification of potential cotreatments to be used with regorafenib for synthetic lethality approaches.

## 3. Mechanisms of Resistance to Kinase Inhibitors

As reported in the previous sections, the clinical outcome of glioblastoma patients treated with kinase inhibitors is not satisfactory. This can be ascribed to both general reasons, dealing with the peculiar features of glioblastoma, and to specific reasons dealing with TKIs.

The first obstacle for glioblastoma therapy is represented by the BBB, a physical barrier that allows the crossing of lipophilic molecules with low molecular weight (<500 Da). Delivery is a major challenge in glioblastoma therapy. Although glioblastoma can breach the BBB, a clinically significant tumor burden has been observed with an intact BBB [92]. In addition, a further factor that influences the overall BBB permeability is the presence of brain efflux pumps, responsible for the active extrusion of drugs out of the brain parenchyma. Several strategies to increase BBB permeability to overcome the issue of drug delivery to brain tumors have been studied [93], including chemical disruption [94], focused ultrasound [95], and inhibitors of efflux transporters [96]. Nevertheless, BBB permeability still remains a key point in therapy effectiveness.

A second crucial feature of glioblastoma, which plays a central role in its resistance to therapy, is represented by its paradigmatic intra- and inter-tumor heterogeneity [97]. This diversity stems from clonal evolution, when accumulated mutations drive the development of distinct subclones influenced by the tumor microenvironment, and from the presence of GSCs, which initiate and sustain tumor growth. GSCs are the tumor cell subpopulation accounting for therapy resistance via mechanisms like enhanced DNA damage responses and the activation of signaling pathways (e.g., Notch, NF-κB, and Wnt/β-catenin). Moreover, glioblastomas often present mixed molecular profiles within a single tumor, leading to variable therapeutic responses. Overall, different subtypes of glioblastoma with distinct molecular profiles co-exist within the same tumor and likely exhibit a differential therapeutic response. Overall, this vast variability is a fundamental glioblastoma characteristic which largely explains why it is not feasible to consider a universally effective therapy for glioblastoma.

Besides general reasons related to glioblastoma peculiarities, some additional features might explain TKIs resistance mechanisms, which are specific to TKIs. Multiple complex factors lead to TKIs resistance. The following four primary mechanisms of escape have been identified: specific mutations, simultaneous activation of pathways, adaptive responses, and the initiation of alternative pathways. Early resistance to therapy in glioblastoma is directly related to specific mutations in genes different from those found in other neoplasms, like lung or gastrointestinal carcinomas. For instance, EGFR antagonists, which are effective in lung carcinoma (as stated before) due to mutations of the kinase domain, have been disappointing in glioblastoma, where mutations of the extracellular domain are largely implicated. In the same way, PDGFR and KIT inhibitors, which are effective in gastrointestinal stromal tumors, are not as effective in glioblastoma because of different mutational patterns. A second mechanism of glioblastoma escape from TKIs is the concomitant activation of multiple pathways, which confers resistance to single-agent therapy. The activation of three different RTKs has been described in glioblastoma in distinct tumor cell subpopulations [98]. The third mechanism is their adaptation to cancer therapy, which has been widely described in cancer. TKI adaptation in glioblastoma has not been fully characterized yet. However, a remarkable adaptation phenomenon has been described in EGFRvIII glioblastoma cells [99]. Resistance to EGFR TKIs occurs by the elimination of mutant EGFRs from extrachromosomal DNA. After TKI withdrawal, clonal EGFR mutations on extrachromosomal DNA resurfaced, demonstrating a powerful cancer cell strategy to evade drug toxicity.

## 4. Possible Strategies to Escape Resistance to TKIs

Overall, all the issues listed in the previous section contribute to lowering the effectiveness of therapies based on a single drug. Although bypass strategies to circumvent the anti-tumor activity of TKIs, based on the activation of alternative pathways, have been described both for mono and combinatorial therapies [100,101], combination therapy has gained considerable attention in recent years and still represents a real therapeutic possibility. The idea is to combine drugs with different targets in search of an additive or synergistic effect. An interesting possibility within the framework of combination therapies is the targeting of the metabolic vulnerabilities characteristic of glioblastoma [102]. Glioblastoma has an increased demand for energy supply and shows relevant metabolic alterations, ranging from increased fatty acid uptake and oxidation, enhanced cholesterol metabolism, and reliance on glucose metabolism. On this path, Aldaz and colleagues recently demonstrated that targeting lipid metabolism might result in improved outcomes of TKI-based therapies using ponatinib, a PDGFR inhibitor [103].

An on-the-edge approach to cancer therapy is immunotherapy. With respect to other cancers, immunotherapy effectiveness has some major limitations in glioblastoma, mainly because of the presence of the BBB. Some other factors contribute to limited immunotherapy efficacy in glioblastoma, mainly multifactorial immunosuppression occurring in the microenvironment and the poor knowledge of the neuroimmune system. Notwithstanding, the concept that immunotherapy in combination with standard care treatments might eventually result in improved therapies is currently under investigation [104,105]. The possibility of combining immunotherapy with TKIs deserves future investigations.

Finally, as a general comment, a crucial issue to be considered when talking about combination therapies is toxicity. Due to the presence of the BBB, targeting brain tumors requires high drug concentrations, and the concomitant use of different chemicals might overall result in excessive toxicity. The strategy of combining drugs with non-overlapping toxicities, decreasing the concentration of each single drug, may be the correct strategy to meet the needs of safety and effectiveness.

## 5. Discussion

The development and implementation of KIs, starting at the end of the last century, represented a breakthrough in cancer therapy with a substantial improvement in the prognosis for many types of cancer. Unfortunately, this does not apply to glioblastoma. As previously stated, currently there are 80 KIs, mostly TKIs, which are in use for a range of tumors. A number of TKIs were tested for glioblastoma treatment, but none of them showed convincing therapeutic efficacy in phase III trials. Tumor resistance to TKIs is a common finding [106]; however, it is not yet clear why inhibitors that are efficacious in other tumors perform so poorly in glioblastoma. Of note, the problem is not limited to KIs but involves other drugs, as discussed, for instance, by the authors of [107]. Recently, a meta-analysis of randomized controlled clinical trials for glioblastoma has been published [108]. In the study, twelve clinical trials were examined, including primary as well as recurrent glioblastomas. A detailed description of these studies can be found in the above-mentioned publication, but the overall take home message is that no clinical improvement was observed in PFS and OS, neither in primary nor in recurrent tumors. There are at least three explanations for the exceptional resistance of glioblastoma to different treatments. We have previously described how the BBB, the over-expression of pumps, and an unfunctional vasculature may prevent drugs from reaching the effective concentration within the glioblastoma tumor. We have also mentioned that cellular heterogeneity may contribute to glioblastoma cell resistance to drugs. Importantly a major source of heterogeneity is the co-existence in the same glioblastoma of different types of GSCs. GSCs were described more than twenty years ago [109,110,111], and since then thousands of papers have been published on this topic. Initially, it was assumed that, similarly to normal neuronal stem cells, GSCs are a homogenous cell population capable of self-renewal and differentiation into the diverse cell types that compose glioblastoma. However, recent experiments, largely relying on single-cell sequencing, suggest that different types of GSCs co-exist in single glioblastoma and that they may be responsible for the post-surgery recurrence of glioblastoma and for the acquired resistance to therapy (for a recent review, see for instance [112]). Of note, GSCs may alter their microenvironment and promote glioblastoma progression [113,114]. GSCs appear to be ideal targets for glioblastoma therapy but, unfortunately, they are particularly resistant to many pharmacological treatments and they also exist in a quiescent state [115].

A further mechanism of glioblastoma resistance to therapy is the structural and functional organization of glioblastoma cells in different kinds of networks. Glioblastoma cells often connect with each other via long protrusions and GAP junctions, structures that protect glioblastoma cells from radiotherapy and are involved in brain invasion [116]. Furthermore, glioblastomas acquire robustness and drug resistance through physical and functional interactions with neuronal cells [117,118] (for recent reviews see [119,120]). It was shown [120] that the secretion of paracrine factors from neurons, as well as the formation of glutaminergic synapses between neurons and glioma cells, drives tumor progression and invasion. A recent proteogenomic analysis which compares pairs of primary versus recurrent tumors [121] demonstrated that the evolution of glioblastomas under the pressure of therapy results in the deactivation of EGFR signaling and the acquisition of signaling involved in neuronal function and synapse formation (both pre- and post-synaptic structure). In brief, primary glioblastomas showed a high level of proteins (and phospho-proteins) involved in cell division, mRNA processing, mitosis, and DNA damage repair driven by EGFR signaling. On the other side, glioblastomas that recur after therapy increased the amount and activation (phosphorylation) of proteins involved in synaptic formation, GTPase activity, and oxidative phosphorylation driven by the activity of RAS, BRAF, and MAPK proteins. Coherently, BRAF inhibitors reduced cellular motility and invasion. Importantly, BRAF inhibitors plus temozolomide increased the survival in murine models of human glioblastoma. Two recently published papers address the issue of elucidating and eventually targeting the glioblastoma cells–neurons connections [122,123]. Both papers used similar approaches, i.e., retrograde rabies virus tracing of human glioblastoma organoids or patient-derived glioblastoma spheroids, to visualize and manipulate the glioblastoma–neurons connectome. The aggregated conclusion is that (*i*) synaptic connections concern different populations of neurons (acetylcholine, glutamate, dopamine, and ATP appear to be the main functional transmitters, while GABA, serotonin, and glycine failed to elicit significant responses in glioblastoma cells), (*ii*) synaptic connections are functional to glioblastoma progression, and (*iii*) feedback to neurons by glioblastoma cells may explain cognitive dysfunctions, sleep disturbances, and seizures.

## 6. Future Perspectives

The take home lessons from these studies are that (1) KIs which target the main drivers of glioblastomas are insufficient to provide a functional long-lasting therapy. Future research should be focused on understanding the mechanisms of acquired resistance for producing combinatory treatments of glioblastoma or preventive therapies to be used during the remission period. (2) Glioblastoma resistance is not driven only by cell-autonomous mechanisms but depends also on interactions with other types of cells in the local environment. As a corollary, new experimental models which take into account these interactions are necessary. One possible approach is based on brain-organoid-based models (see for instance [124]). A further possible approach will be the use of patient-derived organoids which faithfully reproduce the ecosystem of the affected individual, as recently described in [125].

## Abbreviations

KI	Kinase inhibitors
MKI	Multi kinase inhibitor
RTK	Receptor tyrosine kinase
VEGF	Vascular Endothelial Growth Factor
TTFields	Tumor Treatment Fields
PKA	Cyclic-AMP-dependent protein kinase
EGFR	Epidermal Growth Factor Receptor
FGFR	Fibroblast Growth Factor Receptor
EGFR	Epidermal Growth Factor Receptor
NSCLS	Non small cell lung cancer
TGF-α	Transforming Growth Factor alpha
HB-EGF	Heparin-binding EGF-like Growth Factor
MAPK	Mitogen-Activated Protein Kinase
NSCLC	Non Small Cell Lung Cancer
OS	Overall Survival
BBB	Blood–Brain Barrier
PDGF	Platelet-Derived Growth Factor
PDGFR	Platelet-Derived Growth Factor Receptor
VEGFR1-3	Vascular Endothelial Growth Factor Receptors 1, 2 and 3
TIS	Therapy-Induced Senescence
GSC	Glioma Stem Cell
ROS	Reactive Oxygen Species
ATM	Ataxia Telangectasia Mutated
HIF 1 α	Hypoxia inducible factor 1α
CDKN1A	Cyclin dependent kinase inhibitor 1A
FFPE	Formalin fixed paraffin embedded
PSAT1	Phosphoserine aminotransferase 1
